# Vitamin D Sources, Metabolism, and Deficiency: Available Compounds and Guidelines for Its Treatment

**DOI:** 10.3390/metabo11040255

**Published:** 2021-04-20

**Authors:** Ligia J. Dominguez, Mario Farruggia, Nicola Veronese, Mario Barbagallo

**Affiliations:** Geriatric Unit, Department of Medicine, University of Palermo, Via del Vespro 141, 90127 Palermo, Italy; mario.f.med@gmail.com (M.F.); nicola.veronese@unipa.it (N.V.); mario.barbagallo@unipa.it (M.B.)

**Keywords:** vitamin D, cholecalciferol, calcifediol, calcitriol, bone, fracture, osteoporosis

## Abstract

Studies on vitamin/hormone D deficiency have received a vast amount of attention in recent years, particularly concerning recommendations, guidelines, and treatments. Moreover, vitamin D’s role as a hormone has been confirmed in various enzymatic, metabolic, physiological, and pathophysiological processes related to many organs and systems in the human body. This growing interest is mostly due to the evidence that modest-to-severe vitamin D deficiency is widely prevalent around the world. There is broad agreement that optimal vitamin D status is necessary for bones, muscles, and one’s general health, as well as for the efficacy of antiresorptive and anabolic bone-forming treatments. Food supplementation with vitamin D, or the use of vitamin D supplements, are current strategies to improve vitamin D levels and treat deficiency. This article reviews consolidated and emerging concepts about vitamin D/hormone D metabolism, food sources, deficiency, as well as the different vitamin D supplements available, and current recommendations on the proper use of these compounds.

## 1. Introduction

In recent decades, interest in vitamin D has increased exponentially, particularly as a vitamin D deficit has been associated with multiple diseases [1,2], and, globally, there appears to be a high vitamin D deficiency [3]. Currently, the role of vitamin D as a hormone has been confirmed in numerous physiological and pathophysiological processes, related to various organs and systems of the human body [4].

Despite solid evidence concerning the skeletal effects of the vitamin D hormone, at all ages, there are animated discussions about the possible extra-skeletal benefits of vitamin D supplementation [1], and the possibility that high doses could be harmful [5,6]. Nevertheless, most researchers agree that patients who have a vitamin D deficiency (or insufficiency) should receive therapy in order to maintain bone health and overall good health. This is particularly true in patients at high risk of deficiency, such as older adults (particularly those living in long-term care facilities), patients with diabetes, chronic kidney disease (CKD), and malabsorption, among others [7].

Although various guidelines recommend against supplementation with vitamin D for the primary prevention of fractures in community-dwelling, postmenopausal women [8], in patients who already have experienced fragility fractures (secondary prevention), it is essential to obtain adequate serum concentrations of 25-hydroxyvitamin D [25(OH)D] (greater than 30 ng/mL) before starting antiresorptive or osteo-forming treatments, in order to maximize their effectiveness and to avoid hypocalcemia [9,10,11].

While there is growing awareness about the consequences of vitamin D deficiency, information on this deficiency is ambiguous and not sufficient. A general disregard of vitamin D deficiency occurs in spite of its high frequency, the ease of identifying it, and the simple, effective, and inexpensive means available to correct it.

In this narrative review, we summarize the concepts surrounding vitamin D/hormone D metabolism and its food sources; we also explore what is currently known about vitamin D deficiency and the guidelines and molecules available for its correction.

## 2. Vitamin D Sources and Metabolism

Since the identification of the chemical structure of vitamin D in 1930 by the Nobel Prize laureate Adolf Otto Reinhold Windaus, based on the knowledge acquired by several scientists who preceded him [12], there has been extraordinary advances in vitamin D research. Initially, the research focused on bone metabolic effects, recognizing the fundamental role of vitamin D and its metabolites in calcium homeostasis and bone metabolism. Afterwards, with the discovery of 25(OH)D in 1968 [13,14], and successively of 1,25-hydroxyvitamin D [1,25(OH)_2_D] [15,16], the studies expanded to other fields, including immune-mediated diseases, infections, cancer, and cardiovascular diseases [17]. Vitamin D is involved in the mechanisms of regulating the immune system; it regulates the actions of the suppressor T lymphocytes, the synthesis of cytokines, and acts by modulating the processes of cellular apoptosis [18]. Notably, vitamin D also stimulates the intestinal absorption of phosphate and prevents its renal excretion. Thus, the role of vitamin D in bone health is well known, but it represents only one aspect of the pleiotropic functional profile of the molecule.

### 2.1. Sources

The main source of vitamin D is cutaneous synthesis. Contribution from food sources is less prominent because foods containing vitamin D are generally not a daily part of most dietary patterns (Table 1). That is why it is often necessary to prescribe vitamin D supplements to persons who are experiencing vitamin D deficiency due to limited sun exposure, or when cutaneous vitamin D synthesis decreases (e.g., in older adults).

**Figure 1:**
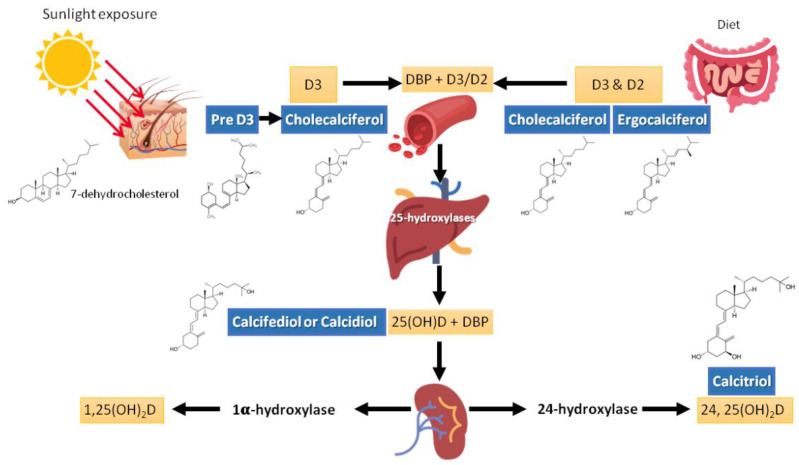
The synthesis of vitamin D3 (cholecalciferol, D3) occurs at the skin where pro-vitamin D3 (7-dehydrocholesterol) is converted to pre-vitamin D3 in response to sunlight exposure (ultraviolet B radiation). Vitamin D3, obtained from the isomerization of pre-vitamin D3 in the epidermal basal layers, or intestinal absorption of natural and fortified foods and supplements D2 (ergocalciferol) and D3, binds to vitamin D-binding protein (DBP) in the bloodstream, and is transported to the liver. D2 and D3 are hydroxylated by liver 25-hydroxylases. The resultant 25-hydroxycholecalciferol [25(OH)D] (calcifediol or calcidiol) is 1-hydroxylated in the kidney by 1α-hydroxylase. This yields the active secosteroid 1,25(OH)_2_D (calcitriol), which has different effects on various target tissues. The synthesis of 1,25(OH)_2_D from 25(OH)D is stimulated by the parathyroid hormone and suppressed by calcium, phosphate, and 1,25(OH)_2_D itself.

Previtamin D3 is synthesized at a cutaneous level from 7-dehydrocholesterol (provitamin D) during exposure to ultraviolet rays of sunlight (wavelengths 290–320 nm). Previtamin D3 is thermally unstable and isomerizes into vitamin D3 (cholecalciferol) after a rearrangement of the triene structure of the molecule [17] (Figure 1). Exposure to UV radiation, amounting to 25% of the minimum erythematic dose (MED) over about a quarter of skin surface (face, hands, and arms) would produce the equivalent of 1000 IU of vitamin D [19]. Fifteen minutes of sun exposure at midday over the entire body during the summer (approximately 1 MED) is the equivalent of 10,000 IU (250 μg) of cholecalciferol [20]; sun exposure of arms, hands, and the face to a dose representing a third or a sixth of the MED produces an equivalent of 200 to 600 IU cholecalciferol intake [21]. However, several factors can affect the efficiency of this synthesis. For example, age, skin color (melanin content), season, weather, latitude, altitude, time of day, clothing, body surface area exposed, holiday habits, use of sunscreen, and skin type (e.g., aging decreases the capacity to synthesize vitamin D in the skin) [22,23,24].

There is no consensus on what constitutes as safe and effective exposure to sunlight for the general population [25], and attempting to provide absolute guidance seems unwise. Specifically, biologically effective radiation is significantly reduced in winter (up to 4-fold) and is greater in the hours closer to noon. Depletion of the atmosphere’s ozone layer can contribute to increased vitamin D synthesis, but also a higher risk of developing skin cancer [24]. On the contrary, the projections of a global ozone layer recovery by the end of the 21st century, assuming continuing compliance with the Montreal Protocol, will have the opposite effect [26]. Nevertheless, although exposure to even low doses of solar simulated UV radiation increases 25(OH)D concentrations, there is great variability in the response, as mentioned, due to various factors. Sunscreen and increased skin pigmentation (i.e., higher melatonin content) can reduce cutaneous vitamin D3 synthesis by up to 90%. In older adults, vitamin D cutaneous synthesis can be reduced by up to 75%, especially during winter and in northern latitudes. Moreover, in old age, renal hydroxylation is decreased. Therefore, it should be taken into account that a patient with optimal values of vitamin D during the summer may present values in the range of deficiency during the winter. Prolonged exposure to sunlight would not produce toxic amounts of vitamin D because of the photoconversion of previtamin D3 to lumisterol and tachysterol, which have no known endocrine functions, as well as to the photoconversion of vitamin D3 itself to suprasterols I and II [27]. The amount of melanin in the skin, by absorbing UV irradiation, can reduce the effectiveness of sunlight in producing vitamin D3. This helps to explain why individuals of the black race and Hispanics living in temperate latitudes have lower 25(OH)D levels. Increasing melanin production by sunlight exposure provides another mechanism by which excess vitamin D3 production can be prevented. However, prolonged UVB and UVA exposure leads to sunburn and DNA damage [24,28].

As shown in Table 1, food sources of vitamin D are mainly of animal origin (cholecalciferol); one could also obtain vitamin D from mushrooms, i.e., of plant origin (ergocalciferol), although there is a stark difference (about 10-fold) between raw mushrooms and those exposed to UV light. Because vitamin D is fat-soluble, it requires bile salts for its absorption, which occurs mainly in the duodenum, successively forming micelles and chylomicrons for its transport. This helps to explain the high frequency of vitamin D deficiency in patients with malabsorption diseases, such as inflammatory bowel diseases, pancreatic insufficiency, celiac disease, cystic fibrosis, cholestatic liver diseases, and short bowel syndrome [29,30,31,32]. Vitamin D content in food is fairly stable, but, as mentioned, the contribution is low because vitamin D-rich foods are not usually consumed frequently. The richest food sources of vitamin D are fatty fish, eggs, sun-exposed mushrooms, liver, and other offal. In some countries, the main sources of dietary vitamin D are fortified foods, of which, milk (cow or of vegetable origin), butter, margarine, and breakfast cereals, enriched with either ergocalciferol or cholecalciferol, are mainly used. The option of fortifying food with vitamin D seems useful, but its consumption is highly variable. Hence, its contribution to reducing vitamin D deficiency is uncertain.

A recent study developed a food frequency and lifestyle questionnaire (FFLQ) to assess vitamin D intake in athletes; the study took place across all seasons; FFLQ was utilized to estimate vitamin D intake compared to intake estimated by food records. Researchers found that serum 25(OH)D was neither associated with the FFLQ-estimated vitamin D intake nor with the estimated vitamin D intake by food records. Conversely, researchers observed a significant association of serum 25(OH)D with tanning bed use in the spring, supplement use in the fall, and BMI across all seasons. These results indicate the influence of factors—other than diet—on the serum concentrations of calcifediol [33]. Because the main dietary sources of vitamin D are of animal origin, a recent simulation study estimated the dietary shifts necessary to optimize vitamin D intake and minimize the carbon footprint. The baseline diet provided approximately one -fifth of adequate vitamin D intake from natural food sources and fortified foods. Optimizing these food sources was linked to an increase in estimated carbon emissions and calorie intake of 3-fold and 2-fold, respectively. When vitamin D-fortified bread, milk, and oil were added as dietary options, along with an increase in fish, and a decrease in sugar, snacks, and cake, adequate vitamin D intake (and other nutrient intakes) were fulfilled within the 2000 kcal/day limit, along with a relatively unchanged carbon footprint. Vitamin D intake goals and carbon footprint reduction by 10% were possible only when compromising on the popularity of the diet [34]. Nevertheless, these are simulated scenarios, for which actual data are still necessary to verify the estimates.

### 2.2. Metabolism

The term vitamin D is generic, as it refers to a group of fat-soluble compounds with a main chain of cholesterol rings; 25(OH)D (calcifediol or calcidiol) has a half-life of two to three weeks and is the main circulating compound, while 1,25(OH)_2_D (calcitriol) has a half-life of only four to eight hours and is the active compound, which interacts with the vitamin D/hormone D receptor (VDR) to exert its physiological function, and regulate its own level via a negative feedback mechanism [35]. Plants and animals have produced vitamin D almost from the time life began on earth. The capacity to metabolize and transport vitamin D to more active forms evolved, as the structures of animals and plants turned out to be more complex, and the cells within these organisms developed more specialized functions. In higher-order animals, the vitamin D receptor (VDR) is found in nearly every cell, and the ability of the cell to produce 1,25(OH)_2_D is also widely distributed [36].

**Figure 2:**
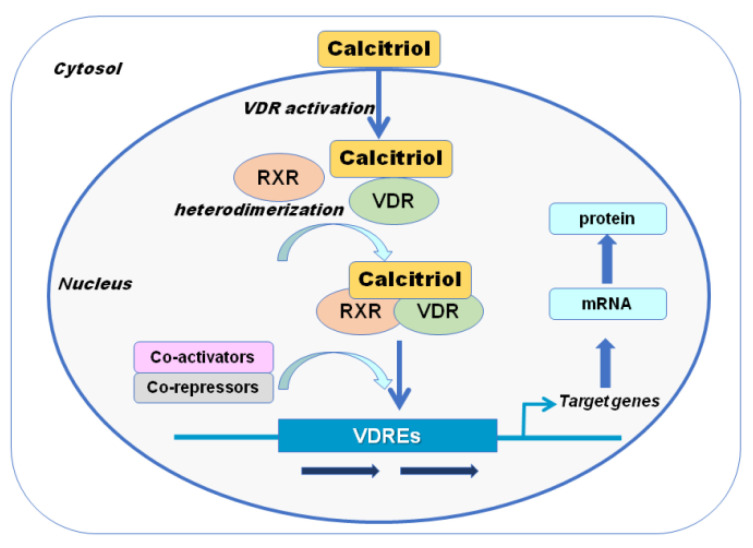
Vitamin D receptor (VDR) action at target cells. Intracellular calcitriol [1,25(OH)_2_D] binds to the VDR; thus, causing its dimerization with the retinoid X receptor (RXR). The ligand-bound VDR–RXR complex binds to structurally distinct vitamin D response elements (VDREs) in multiple, widely spaced vitamin D-responsive regions, and this causes a modification in the recruitment of co-activators or co-repressors, which leads to positive or negative transcriptional regulation of gene expression.

The VDR is a member of a large family of proteins that includes the receptors for the steroid hormones, thyroid hormone, vitamin A family of metabolites (retinoids), and a variety of cholesterol metabolites, bile acids, isoprenoids, fatty acids, and eicosanoids. VDR was first described in 1969 [37] as a binding protein for an at that time unknown vitamin D metabolite, subsequently identified as 1,25(OH)_2_D). VDR was then cloned and sequenced in 1987 [38,39]. Experimental models with knocked-out VDR exhibited the full phenotypes of severe vitamin D deficiency, indicating that VDR was the major mediator of vitamin D action [40]. VDR is extensively, although not universally, distributed throughout different tissues of the human body [36,41]; 1,25(OH)_2_D initiates or suppresses gene transcription by binding to the VDR, which triggers hetero-dimerization of VDR with the retinoid X receptor. The heterodimer then translocates to the nucleus, where the complex binds to vitamin D response elements and alters gene transcription [36] (Figure 2).

Once vitamin D (2 and 3) reaches the circulation, it is weakly bound to the vitamin D binding protein (DBP) for transport and is stored in adipose tissue. It is then metabolized into 25(OH)D, mainly in the liver, thanks to the action of various hydroxylases, including cytochrome P450 (CYP)2R1 and CYP27A1, but this can occur in a variety of tissues in autocrine/paracrine modality [35]. The rate of conversion to 25(OH)D may be slower in people receiving high doses of vitamin D [42]. Afterwards, hydroxylation occurs in the renal tubule to produce the active molecule (1,25(OH)_2_D [35,43]. At least two proteins, cubilin and megalin, facilitate the entry of DBP-25(OH)D complex through the renal tubule cellular receptors. The reduction of these proteins leads to a urinary loss of 25(OH)D and, consequently, to its deficiency. Renal tubular cells contain 2 hydroxylases that are part of the cytochrome P450 system: 1-alpha-hydroxylase (CYP27B1) and 24-alpha-hydroxylase (CYP24A1), which, by hydroxylating 25(OH)D, produce the active form of vitamin D (1,25(OH)_2_D-calcitriol) or the inactive metabolite 24,25(OH)_2_D [44,45]. Noteworthy, various steps in vitamin D metabolism, such as the binding of vitamin D3, D2, and 25(OH)D to their transport protein (DBP) as well as the liver and renal hydroxylation enzymes to produce 25(OH)D and 1,25(OH)_2_D, depend on magnesium as a cofactor; hence, in the presence of magnesium deficit, transport and activation of vitamin D would be blunted [46]. Magnesium also plays a critical role in parathyroid (PTH) synthesis and release, which are inhibited in magnesium-depleted states [47,48,49,50]. Furthermore, low dietary magnesium intake may alter PTH response to 25(OH)D [51]. Thus, the deficit of each of these compounds, magnesium, and vitamin D, feeds the deficit of the other, which may lead to a perverse cycle with further worsening of both deficits. The combined effects of magnesium and vitamin D deficiency may lead to clinically relevant outcomes, such as a higher risk of fragility fractures, particularly in women [52]. In fact, it is plausible that similar harmful effects of this detrimental combination could be observed in other major clinical outcomes. A large study by Deng et al. [53] investigating potential interactions between vitamin status, magnesium intake, and mortality found that high total magnesium intake was independently associated with reduced risk of 25(OH)D deficit (<12 ng/mL) or insufficiency (12–20 ng/mL). An inverse association of serum 25(OH)D with mortality (particularly due to cardiovascular disease and colorectal cancer) was modified by high magnesium intake (i.e., the inverse association was primarily present among those with magnesium intake above the median). Similarly, a recent nested double-blind RCT, within the Personalized Prevention of Colorectal Cancer Trial, evaluating whether magnesium supplementation affects vitamin D metabolism, involving 180 participants showed that an optimal magnesium status was related to improvement of the 25(OH)D status [54].

Studies in human kidneys show that, in contrast to those in experimental models, the distal nephron is the main site of expression of 1-alpha-hydroxylase [55]. The circulating concentration of 1,25(OH)_2_D depends on the availability of 25(OH)D and on the activity of 1-alpha-hydroxylase and 24-alpha-hydroxylase. The regulation of 1-alpha-hydroxylase activity depends mainly on the concentration of PTH, calcium, phosphorus, and fibroblast growth factor 23 (FGF23) [35,43,56]. FGF23 limits the activity of 1-alpha-hydroxylase, thereby inhibiting the renal production of 1,25(OH)_2_D, while simultaneously increases the production of 24-alpha-hydroxylase and 24,25(OH)_2_D [56,57]. 1,25(OH)_2_D stimulates FGF23 which reduces renal phosphate reabsorption counteracting the increase in gastrointestinal absorption induced by 1,25(OH)_2_D [57]. Both the active hormone 1,25(OH)_2_D and its precursor 25(OH)D are, in part, degraded by 24-hydroxylase. The activity of this enzyme, in turn, is stimulated by 1,25(OH)_2_D and decreased by the elevation of PTH [35,58].

As mentioned, 1-alpha-hydroxylase is also expressed in other tissues besides the kidney, such as the gastrointestinal tract, vascular tissue, breast, skin, osteoblasts, and osteoclasts [59]. That is why some diseases, i.e., sarcoidosis, can manifest with hypercalcemia where there is an increased production of 1,25(OH)_2_D by pulmonary macrophages and lymph nodes [60]. Table 2 shows the main biological actions of vitamin D/hormone D.

### 2.3. Pharmacokinetics

Results from clinical studies investigating the dose–response curve to vitamin D are markedly variable, attributable to various dosing regimen, administrative routes, assay methods for measuring 25(OH)D, demographics, and also regulation of endogenous vitamin D production, which, as mentioned above, also depends on several factors. There is lack of agreement as to the optimal level and no consensus as to the dose that will bring individual patients to that level (see next sections). Aloia et al. performed a 6-month, prospective, double-blinded, double-dummy, randomized, placebo-controlled trial (RCT) of vitamin D3 supplementation adjusting vitamin D intake every 2 months, aiming to determine the intake of vitamin D3 needed to raise serum 25(OH)D to >30 ng/mL. After two dose adjustments, almost all participants attained concentrations of 25(OH)D >30 ng/mL with a mean daily dose of 3440 IU. The use of computer simulations predicted an optimal daily dose of 4600 IU to obtain that most participants would be within the range of 30–88 ng/mL. No hypercalcemia or hypercalciuria were observed. They concluded that determination of intake required to attain optimal serum 25(OH)D concentrations must take into account the wide variability in the dose–response curve and basal 25(OH)D concentrations [61]. Pharmacokinetics of the distribution of vitamin D and its metabolites must consider absorption, distribution, metabolism, and excretion, in addition to the diverse routes of administration. As such, intestinal absorption is altered in malabsorption syndromes, by interaction with medicaments, and by genetic causes; distribution is modified depending on storages in fat and skeletal muscle; metabolism, as well as excretion, are altered in liver and kidney disease. The 25-hydroxylase CYP3A4 enzyme, which converts ergo- and cholecalciferol to 25(OH)D, is a phase I biotransformation enzyme for many drugs. A number of drugs are metabolized by CYP3A4, while other medications may inhibit or induce CYP3A4 activity [62]. Table 3 shows drugs that interact with vitamin D absorption, metabolism, and side effects (Table 3).

In particular conditions, such as those of HIV-infected patients in whom vitamin D deficiency is prevalent, a study investigated the pharmacokinetics of 25(OH)D, the effect of antiretroviral treatment and others factors that may influence the pharmacokinetics, and vitamin D3 dosing scheme to reach the 30 ng/mL. Among 422 HIV-infected patients 25(OH)D pharmacokinetics were best described by a one compartment model with an additional endogenous production. The effects of season and skin phototype were significant on production rate. The endogenous production was 20% lower in non-white skin phototype patients and was decreased by 16% during autumn, winter, and spring. No significant differences in 25(OH)D concentrations were related to antiretroviral drugs. To obtain concentrations between 30 and 80 ng/mL, the dosing recommendation was 100,000 IU every month [63].

Because the pharmacokinetics of vitamin D is complex and depends on a number of determinants, new mathematical models have been proposed in the attempt to better predict the response to different existing compounds and also to new metabolites in development [62,64].

There are several differences in the pharmacokinetic characteristics of the diverse vitamin D compounds and activated forms used to treat vitamin D deficiency. This will be discussed in the section Management of vitamin D deficiency below.

### 2.4. Measurements

The circulating concentration of 25(OH)D is currently accepted as the best marker of vitamin D status, and has been used by various national and international organizations for establishing vitamin D dietary requirements and for population surveillance of vitamin D insufficiency or deficiency [7,65,66]. As mentioned, 1,25 (OH)_2_D has a very short half-life, its circulating concentration is low, and it is constantly modifying due to a tight regulation. Furthermore, in states of genuine vitamin D insufficiency, 1,25 (OH)_2_D levels may be normal due to the compensatory increase in PTH, the main regulator of renal 1-alpha-hydroxylase and, consequently, the optimal vitamin D levels are generally considered to be those that maintain PTH within the normal range [7,17,65,66]. In fact, elevated PTH values could be considered an indicator of vitamin D insufficiency.

Nevertheless, concentrations of 25(OH)D has been historically indicated as having slight physiologic regulation, hence other measures have been suggested as indicators of vitamin D status. For example, there are current discussions regarding the possibility of considering free 25(OH)D, i.e., unbound to transporter proteins, or even the ratio of 24,25(OH)_2_D:25(OH)D [67].

When comparing laboratory analytical methods measuring 25(OH)D, differences of at least 10–15% are found, suggesting that caution is required when comparing different methods. In addition, immunoassays do not always distinguish between 25(OH)D3 and 25(OH)D2. The first assays developed used in-house competitive binding assays. Afterwards, an iodinated tracer was introduced in the 1990s, leading to the development of a commercial radiommunoassay. In 2007, fully automated immunoassay procedures were introduced. In order to get over the limitations of the automated methods, liquid chromatography/mass spectrometry assays (LC–MS/MS) were increasingly adopted. The LC–MS/MS assay of the U.S. National Institute of Standards and Technology (NIST) was accepted as the reference measurement procedure, introducing the standard reference material for vitamin D in 2009 [68]; the vitamin D standardization program was established in 2010.

However, the quality of 25(OH)D measurement methods is extremely variable, an issue still broadly present and that hampers the evaluation of currently used guidelines quality. Likewise, there is large uncertainty on the quality of free 25(OH)D quantification, which limits its comparative evaluation with 25(OH)D. There are standardization programs such as the ‘Vitamin D Standardization Program’ (VDSP) and ‘Vitamin D External Quality Assurance Scheme’ (DEQAS). The latter, supported by CDC-standardized target values, has examined quarterly the performance of 700–1000 laboratories carrying out 25(OH)D for thirty years, verifying problematic assays and kit manufacturing companies [69]. In the U.S., the National Institute of Health office of Dietary Supplements is financing the development of a reference method for 1,25(OH)_2_D, in order to help standardize its values in research contexts and help clarifying its usefulness [65]. The standardization programs are crucial to help elucidate the definition of true vitamin D deficiency. Yet, analytical standardization is not the only challenge that is faced in the quantification of vitamin D metabolites. Patient-dependent variability factors are fundamental and are recognized confounders responsible of inaccurate results (i.e., hemodialysis patients, pregnancy). In addition, conditions that may alter the affinity of DBP to either 25(OH)D3 or 25(OH)D2 in the immunoassays may result in impreciseness of serum 25(OH)D values, for example, in persons using ergocalciferol (D2) supplements [70].

### 2.5. Optimal Values

As with the discrepancies of accurate measurements, there is no consensus on what is the most adequate concentration of 25(OH)D for health, both for the skeleton and for other organs and systems. Although it would appear to exist an ideal value of vitamin D status, this consideration is more complex. In general, a serum concentration of over 20 ng/mL is assumed to be ideal for the general population, and over 30 ng/mL for those older than 65 years old, patients with pre-existing bone conditions under treatment with antiresorptive or anabolic bone-forming agents for the reduction of fragility fracture risk, or under therapy that increases the risk of fragility fractures (i.e., glucocorticoids, anti-hormonal cancer therapies). Nevertheless, national and international agencies indicate diverse ranges of circulating 25(OH)D for considering an ideal vitamin D status, which also implies a different definition for deficiency or insufficiency of this fundamental vitamin/hormone. This is illustrated in Table 4.

At a recent international conference on controversies in vitamin D, most interest groups agreed to categorize vitamin D status in adults as follows: (i) sufficiency, defined as a 25(OH)D concentration >20 ng/mL; (ii) insufficiency, defined as a 25(OH)D concentration between 12 and 20 ng/mL; (iii) deficiency, defined as a 25(OH)D concentration <12 ng/mL; (iv) toxicity risk, defined as 25(OH)D concentration >100 ng/mL in adults consuming considerable amounts of calcium [71].

## 3. Vitamin D Actions

### 3.1. Calcium, Phosphate, and Bone Metabolism

The effects of vitamin D on mineral homeostasis are exerted by modifying the expressions of several genes in the small intestine, kidneys and bone. The activation of VDR by 1,25(OH)_2_D stimulates intestinal calcium and phosphate absorption, renal tubular calcium reabsorption, and calcium mobilization from the bone. Bone mineralization triggered by 1,25(OH)_2_D occurs mainly by increasing intestinal calcium and phosphate absorption to maintain an adequate calcium–phosphate product, which crystallizes in the collagen matrix leading to bone mineralization [4,17]. 1,25(OH)_2_D stimulates the expression of osteocalcin, the main non-collagenous protein in the skeleton. PTH and 1,25(OH)_2_D enhance bone resorption by eliciting the expression of receptor activator of nuclear factor kappa-B (RANK) ligand (RANKL) on osteoblasts cell membrane and releasing it into the circulation. RANKL interacts with RANK on the monocytic osteoclast precursor cell leading to the merging with other monocytic cells and the formation of mature osteoclasts. Bone resorption occurs by the action of osteoclasts that through hydrochloric acid increase the release of calcium into the circulation and of collagenases to remove the collagen matrix. Additionally, 1,25(OH)_2_D directly inhibits PTH production and induces FGF23 production in osteocytes as a part of negative feedback loops to maintain serum calcium and phosphate concentration in a physiologic range [72]. Thus, vitamin D, together with PTH and FGF23, give rise to an endocrine network that plays a crucial role in maintaining calcium and phosphate homeostasis, as well as normal bone growth and mineralization.

### 3.2. Other Non-Skeletal or Mineral Actions

Vitamin D has numerous actions in addition to those strictly related to calcium and phosphate homeostasis and bone metabolism. This can be explained, at least in part, by the fact that VDR is present in most tissues, including the skin, skeletal muscle, endocrine pancreas, immune cells, brain, adipose tissue, breast, vascular tissue, as well as in a number of cancer cells and the placenta [1,2,4,36,41]. Current evidence confirms that VDR activation by 1,25(OH)_2_D produces numerous biological actions in these tissues through genomic and non-genomic pathways, i.e., anti-proliferative and pro-differentiation effects on keratinocytes, immunomodulatory effects on activated B and T lymphocytes and macrophages, anti-metastatic effects on various cancer cells, effects on muscle function, maternal/child health, potential protective effects against cardiovascular diseases, metabolic disorders, and pregnancy complications [1]. All of these direct actions have been primarily studied in preclinical investigations.

Numerous observational epidemiological studies in humans have shown significant associations of low 25(OH)D concentrations with increased (current and future) health risks, in line with the various known actions of vitamin D. However, RCTs, the ‘gold standard’ for assessing the efficacy of treatments, and meta-analyses, have frequently failed to provide supportive evidence for the expected health benefits of vitamin D supplementation [73,74]. Such RCTs have used designs developed for testing drugs while vitamin D is a nutrient, in which a different rational should guide trial designs. Considering that most participants enrolled in the trials did not have vitamin D deficiency, an extra provision will not induce benefits. Contrarily, the fewer participants with vitamin D deficiency at baseline would likely require higher doses in order to achieve optimal values related to health benefit [73]. This is illustrated by the results of a meta-analysis of 25 RCTs, testing whether vitamin D supplementation would decrease the risk of upper respiratory tract infection rates. When participants were stratified by vitamin D status, those with baseline 25(OH)D below 10 ng/mL had a stronger risk reduction when using supplementation on a daily or weekly doses, but not in those receiving large doses, which has been associated with adverse skeletal effects [5,75]. In addition to the numerous small RCTs, the findings of four large trials have been published showing that vitamin D supplementation does not prevent hard-disease endpoints, such as cardiovascular disease, cancer, fractures, or falls, aside from a possible beneficial effect against cancer mortality, although some benefits have been reported for some intermediate outcomes [74].

Other actions with evidence in the literature are those that involve muscle. Vitamin D seems to be indispensable for athletic performance [76]. As mentioned above, muscle is one of the tissues where VDR is present and the expression of multiple myogenic transcription factors enhancing muscle cell proliferation and differentiation is caused by an exposure of skeletal muscles to vitamin D [77]. Some controversies that have arisen regarding inconsistencies in studies investigating the presence of VDR in skeletal muscle [78,79] have been resolved by later studies, providing strong support for the presence of VDR in skeletal myocytes combining multiple techniques [80]. Calcitriol activates multiple metabolic processes in the muscular tissue, resulting in the stimulation of protein synthesis and in an increased number of fast twitch muscle cells (type II fibers), responsible for high power output, fast muscle contraction, and muscle development. Both protein synthesis and increased type II muscle cells lead to the increased muscle contraction velocity and strength [77,81,82]. These effects have led to a number of studies testing the association of vitamin D status with muscle strength and exercise performance in athletes. However, a recent review of the available studies has shown that their results are inconclusive with no clear relationship between serum 25(OH)D levels and performance [83]. Likewise, even if some studies in older populations seem to point to the positive effects of vitamin D supplementation on muscle performance, the results from studies conducted in athletes are inconsistence [83]. The variabilities in the results may be due, at least in part, to the fact that there is a high prevalence of vitamin D deficiency among athletes and the response to supplementation may be different depending on the degree of deficiency or the lack of it, as in the case of respiratory infections mentioned. This topic will be discussed in the next sections.

## 4. Vitamin D Deficiency

Even if, as mentioned, the cutaneous synthesis of vitamin D3 occurs rapidly in the presence of adequate solar UVB exposure, due to human behavior vitamin D deficiency is widespread [84,85]. Indeed, the groups at highest risk include those who lack effective exposure to sunlight (i.e., indoor work, sun avoidance, long-term care residents, etc.). This may be a consequence of various cultural, climatologic, or religious reasons, as well as to skin type and pigmentation. For example, vitamin D deficiency was traditionally considered unusual in Africa, but a systematic analysis of African countries showed that severe vitamin D deficiency is present in as much as 18% of all African persons, with higher prevalence in some groups due to particular cultural/behavioral practices [86,87,88,89,90].

As discussed above, the serum concentration of 25(OH)D is considered the most accurate estimate of vitamin D status [7,65,66,91], despite the difficulties in its standardization. The definition of vitamin D insufficiency or deficiency varies according to diverse national and international agencies (Table 3); nevertheless, a concentration of 25(OH)D below 20 ng/mL is widely used for considering insufficiency, and below 10–12 ng/mL is generally indicative of true deficiency. Despite extreme expressions of morbidity specifically consequent to vitamin D deficiency, such as rickets or osteomalacia, are not very frequent (but still present) in industrialized countries, cases of subclinical deficiencies are very common. The implications of this recurrent milder degree of deficiency, or insufficiency, are not entirely clear, but it is foreseeable that it may contribute not only to a rise in fragility fractures but also to an increase in other highly prevalent morbid processes.

Specific conditions associated with vitamin D deficiency include malabsorption syndromes, such as celiac disease, gastric bypass, short bowel syndrome, and cystic fibrosis [3]. Obese persons are at higher risk of vitamin D deficiency because of reduced availability due to its sequestration in body fat [92]. Medications that induce hepatic enzymes can accelerate the degradation of vitamin D, increasing the risk of vitamin D deficiency, i.e., phenobarbital, carbamazepine, rifampin, spironolactone, dexamethasone, nifedipine, and clotrimazole [93]. Because vitamin D is fat soluble, cholestyramine and orlistat can reduce its absorption and should be taken several hours apart from it [94]. Chronic liver and kidney diseases by impairing the activation of vitamin D can also increase the risk for vitamin D deficiency. The prevalence of patients with vitamin D deficiency is highest in old age, obese, nursing home residents, and hospitalized patients [3].

The consequences of bariatric surgery deserve a special mention, since it is a procedure that is continuously increasing in a world in which obesity is rising exponentially. In particular, vitamin D deficiency and secondary hyperparathyroidism is not infrequent in obese patients, and the deficiency can get worse following gastric bypass surgery [95]. Hence, it is fundamental to recognize and treat vitamin D deficiency before bariatric surgery in order to prevent postoperative complications and metabolic bone disease in the long-term with the consequent increase in fracture risk. The harmful skeletal effects of bariatric surgery are undoubtedly multifactorial, including nutritional factors such as vitamin D deficiency and inadequate mineral intake and absorption [96]. The severity of the deficiency may vary with the various surgical procedures and patient’s characteristics, whereby, a single dose of vitamin D may not meet the needs of all patients [97]. Nevertheless, there is lack of consensus on the dosage and frequency of optimal vitamin D supplementation, before and after the bariatric surgery [98].

It is also important to remember that low circulating levels of 25(OH)D are common during acute illnesses [99]. Several factors may help to explain this finding, including the eventual pre-existing vitamin D deficiency as well as the effects of fluid shifts in the intravascular space that may dilute the actual concentration of 25(OH)D. However, it is not clear whether vitamin D supplementation in intensive care patients is of any benefit. A double-blind RCT that included 475 heterogeneous critically ill patients did not show improvement in hospital length of stay or overall mortality, but demonstrated in a secondary analysis that high-dose oral vitamin D3 supplementation improved mortality in patients with severe vitamin D deficiency [100]. It is possible that higher doses are needed in the critical patient due to the marked release of cortisol, which may impair hepatic and renal hydroxylation of vitamin D [101].

There is compelling evidence that confirm the prominent prevalence of vitamin D deficiency across different athletic disciplines. A comprehensive review on the available studies showed that 32% of basketball professional players were deficient and 47% had 25(OH)D levels in the range of insufficiency; among National American Football League players, 26% had vitamin d deficiency and 42–80% had insufficiency. Similar deficiencies and insufficiencies have been found in most dancers, swimmers, volleyball players, taekwondo fighters, runners, weightlifters, etc. [102]. Particular conditions among athletes may further predispose them to vitamin D deficiency. For example, dark-skinned athletes may be at higher risk and may need ten times longer exposure to UVB radiation to generate the same reserves of vitamin D as light-skinned athletes. A study on professional hockey players found that there were none with vitamin deficiency and only 13% with insufficiency, which was attributable to the fact that 96.2% of the players were Caucasians [103].

Moreover, athletes from regions located north of parallel 35th N, for example Russia and Finland, are particularly at risk because the angle at which sun rays enter the atmosphere becomes more shallow, leading to their dissipation [104]. The decreased solar intensity and cold temperatures discourage skin exposure, especially during the winter, hindering extensively the synthesis of vitamin D in these populations. As such, in a study of 131 Finnish gymnasts and runners, living at latitude 60 degrees north, deficiency in serum concentration of 25(OH)D was found in over 80% of participants [105]. Likewise, a study in male youth Russian soccer players, mean age 15.6 ± 2.4 years, reported levels below 30 ng/mL in 42.8% of participants. In this study, daily supplementation with 5000 IU cholecalciferol for two months was able to increase 25(OH)D levels to a range between 30 and 60 ng/mL in 74% of the sample [106]. A systematic review and meta-analysis of 23 studies comprising 2313 athletes (mean age of 22.5 ± 5.0 years, 76% male) from different disciplines found that 56% of participants had inadequacy of serum 25(OH)D levels (<32 ng/mL), which varied by geographical location and was worst for winter and spring seasons, indoor activities, and mixed sport activities. The risk was slightly higher for latitudes higher than 40 degrees N [107]. According to a recent review, vitamin D supplementation helps improve serum 25(OH)D levels and, in some studies, can positively affect muscle performance, but the results are inconclusive, with several studies showing no effect [83]. Likewise, a systematic review and meta-analysis of thirteen RCTs comprising 433 athletes found that among athletes with baseline serum 25(OH)D levels below 30 ng/mL, vitamin D supplementation with 3000 IU/day and 5000 IU/day led to significant increase at degree latitudes higher than 45, and to sufficiency concentrations during winter months. Only seven out of thirteen included RCTs measured physical performance parameters and overall they did not demonstrate significant effects of vitamin D supplementation during 12 weeks of follow-up. This may be explained, at least in part, by the large heterogeneity of the trials [108]. Thus, well-designed RCTs examining the effect of vitamin D supplementation on serum 25(OH)D levels, physical performance, and injuries in specific sports, latitudes, ethnicities, and baseline vitamin D status are warranted.

There is some evidence suggesting that vitamin D may be important for the prevention of stress fractures in athletic populations [109,110], which are more frequently occurring in the tibia, fibula, femur, and tarsal and metatarsal bones of the foot, and generally attributable to sudden increase in training, decreased lower extremity strength, low BMD, and/or history of menstrual disturbances. Stress fractures have been reported most commonly in military recruits with intensive training programs, in which a serum 25(OH)D lower than 20 ng/mL was prospectively associated with a significant increase in stress fractures [110]. Moreover, British Army recruits with 25(OH)D levels over 20 ng/mL at the time of injury, recovered more quickly from stress fracture injury [111]. Lappe et al. conducted a double-blind RCT involving 3700 female U.S. navy personnel aiming to evaluate the effects of supplementation with cholecalciferol (800 IU/day) plus calcium (2 g/day) on the incident stress fractures. They found a significant reduced risk of stress fractures by up to 20% vs. controls despite the negative influence of certain prevalent lifestyle [109]. There is still no evidence of the effects of vitamin D status and supplementation on stress fractures in elite athletes, which directly affect training and competition. Studies in this area are challenging due to the multiple variables involved other than vitamin D, such as overtraining, smoking, age, poor quality diet, and eventual menstrual disorders.

Interestingly, physical exercise itself can increase vitamin D levels. Some observational studies showed that regular exercise or high physical activity were associated with higher concentrations of serum 25(OH)D, even after adjusting for sun exposure [112,113,114]. Furthermore, one study found that increased physical activity was associated with higher serum 25(OH)D concentrations, not only in the summer, but also in the winter, when sunlight was very limited [115]. All of these are results in chronic conditions. In addition, a study by Sun et al. aimed to examine whether acute endurance exercise had an effect on serum 25(OH)D concentrations in twenty young, active adults. Participants performed a cycling exercise for 30 min at 70% maximal oxygen uptake. Serum 25(OH)D concentrations were significantly increased at 0, at 1, and 3 h after exercise, as well as 24 h later, with greater increases in men when compared to women [116]. Another study involving fourteen adults also found significant increases in serum 25(OH)D concentrations after intensive stretch shortening cycle exercise [117]. Conversely, other studies did not observed any 25(OH)D acute change after exercise in young adults or cyclists [118,119], but the investigators did not consider the time course of circulating 25(OH)D concentrations after endurance exercise in those studies, as opposed to the study by Sun et al.

Therefore, regular monitoring vitamin D status and supplementation in winter, especially for those residing at latitudes where UVB exposure is negligible, or those who practice indoor disciplines, is essential to ensure healthy athletes. This could be useful to identify athletes at high risk for stress fractures during intensive training and treat those with vitamin D deficiency. Maintaining an adequate vitamin D status may also provide additional health benefits for athletes. However, vitamin D supplementation should be individualized, avoiding high doses for athletes who do not need them, without proven benefit, and even with the risk of harm (see below section on Vitamin D Excess/Toxicity).

### 4.1. Dimension of the Problem—Epidemiology

Suboptimal vitamin D status is pervasive worldwide [3,85]. According to a systematic review by Hilger et al. from 2014, 37.3% of the global population had 25(OH)D circulating concentrations below 20 ng/mL, while the variability in vitamin D status across the world was not correlated with latitude. Severe vitamin D deficiency, generally defined as concentrations below 12 ng/mL, was reported in approximately 7% of the population worldwide with considerable variation between diverse countries and populations. Nevertheless, severe vitamin D deficiency occurs in high-risk populations worldwide [85]. A recent report showed that Africa had poor vitamin D status with 34% of the population presenting 25(OH)D lower than 20 ng/mL [120]. However, there are scarce data from Africa and South America, as well as for infants, children, adolescents, and pregnant women worldwide [121].

Analyses of data from the National Health and Nutrition Examination Survey (NHANES) 2005–2006 showed that 41% of US adults had 25(OH)D values below 20 ng/mL [122]. A more extensive analysis of NHANES data from 1988–2010 reported that 14–18% of US adults had 25(OH)D values below 16 ng/mL with significant differences among ethnic groups, with a higher proportion for non-Hispanic blacks (46–60%), when compared to Mexican Americans (21–28%), and to non-Hispanic whites (6–10%) [123]. In Europe, analyses in 14 population studies using standard protocols (VDSP) in a sample of 55,844 European participants showed that, irrespective of age group, ethnic mix, and latitude, 13.0% had 25(OH)D concentrations below 12 ng/mL on average during the year, with variations from 17.7% to 8.3% during winter (October to March) and summer (April to November), respectively. The prevalence of vitamin D deficiency (<20 ng/mL) was 40.4%. Dark-skinned ethnic groups had a 3 to 71-fold higher prevalence when compared to white populations [124]. A study from the UK reported that 46.6% of white adults had 25(OH)D below 16 ng/mL during winter and spring, which decreased to 15.4% during summer and fall. In this population the odds ratio for increased risk of low 25(OH)D was 2.03 for obesity and 2.38 for those living in Scotland vs. those living in southern England [125]. Other studies reported 61% and 23% of UK adults (19–64 years) with serum levels of 25(OH)D below 20 ng/mL and below 10 ng/mL, respectively [126]. In a large sample of UK South Asians (aged 40–69 years), a study showed that 92% had serum 25(OH)D lower than 20 ng/mL, 55% below 10 ng/mL, and 20% below 6 ng/dL [127]. Among 5034 Australian adults, 20% had serum 25(OH)D lower than 20 ng/dL [128].

The prevalence of hypovitaminosis D in developing countries, according to a review by Arabi et al., varies widely, ranging from 30 to 90%, also depending on the various cut-off values used within specific regions, while it is independent of latitude. In China and Mongolia, a high prevalence was reported, especially in children, of whom up to 50% had serum 25(OH)D levels below 5 ng/mL. In countries with ample sunshine throughout the year, such as Sub-Saharan Africa and the Middle East, one-third to one-half of persons had serum 25(OH)D levels lower than 10 ng/mL, according to studies published in the past decade. Risk factors for hypovitaminosis D in developing countries are similar to those reported in industrialized nations [90]. A review of available data from China from 2000 to 2012 reported a high prevalence of 25(OH)D levels below 12 ng/mL (~37%) and below 30 ng/mL (~72%) [129]. Worldwide, newborns, and older adults living in institutions are the age groups with the greatest risk of deficiency [85].

### 4.2. Clinical Characteristics

Osteomalacia literally means “soft bones” referring to the alterations in bone structure, most often caused by severe vitamin D deficiency, which in children results in rickets (the most severe form of osteomalacia) and in clinical and histologic manifestations of osteomalacia in adults (in general milder forms), particularly in older adults [3,17,130,131]. Rickets remain a significant public health concern worldwide, including even highly developed countries [130,132], despite largely funded programs, and the fact that vitamin D deficiency-related rickets is cured by vitamin D administration. Recently, several international professional societies have prepared a memorandum in order to persuade the World Health Organization to start an implementation program in order to eradicate nutritional rickets by 2030 [132].

Rickets results from a defective mineralization in the growing skeleton, while osteomalacia in adults results from reduced skeletal mineralization taking place after the epiphyseal plate fusion [133]. Rickets is an important problem even in countries with adequate sun exposure [130]. Insufficient calcium intake may also cause rickets [134]. The precise, or even estimated, prevalence of osteomalacia in adults caused by vitamin D deficiency is not available, probably because it is often not identified or misdiagnosed as osteoporosis [131]. In fact, bone mineral density (BMD) measurements used widely cannot differentiate between osteoporosis and other metabolic bone disorders, including the different types of osteomalacia. The distinction can be made with bone histomorphometry [135], but this method is not usual in the clinical practice because the necessary skills and training for the performance of transiliac bone biopsy and for its histological evaluation are rarely available; also, because it is an invasive and painful procedure that the patients frequently do not accepted. Some clinical criteria have been recently proposed in order to overcome the difficulties of performing bone biopsies when the patients present with vague clinical manifestations, such as diffuse bone pain and muscle weakness, or some radiological signs (reduced BMD, pseudofractures on X-ray, or diffuse multiple uptakes on bone scintigraphy), and laboratory findings [136]. Nevertheless, these criteria have not been yet validated.

The reduction in calcium and phosphorus absorption (about 80–90% and 40–50%, respectively) due to vitamin D deficiency leads to decreased ionized circulating calcium and, thereby, secondary hyperparathyroidism. PTH maintains calcium levels through mobilizing bone calcium stores by increasing bone resorption and by rising calcium tubular reabsorption; it increases phosphate urinary excretion by inhibiting its renal reabsorption [137]. The clinical hallmark of rickets and osteomalacia is the finding of severe 25(OH)D deficiency, high serum alkaline phosphatase, normal serum calcium, and low to low-normal serum phosphate. The typical generalized defective osteoid mineralization is a consequence of inadequate calcium–phosphate product [138].

Disrupted endochondral bone formation in rickets predominantly affects areas of rapid bone growth (i.e., long bone epiphyses and costochondral junctions). Defective chondrocyte maturation results in cell hypertrophy leading to growth plates widening. Classic rickets signs include rachitic rosary due to costochondral junctions hypertrophy, sternum protrusion, ribs involution, skull deformities, such as craniotabes and frontal bossing, poor growth, lower limb bowing, delayed dental eruption, and enamel defects. Hypocalcemia causing seizures, laryngospasm, tetany, cardiomyopathy, and death can be observed in severe cases [130,138]. Clinical manifestations of osteomalacia in adults are mainly diffuse bone pain and tenderness, muscle weakness, and fragility fractures [3,131,139,140]. However, these symptoms are not specific and can be present in non-skeletal disorders. Detection of pseudofractures (Looser zones) in X-ray is fairly diagnostic of osteomalacia along with other clinical evaluation parameters. The reduced mineral content and consequent diminished bone strength render older adults with vitamin D deficit more susceptible to fragility fractures in both axial and appendicular skeleton [131,141]. In older populations, muscle weakness associated with vitamin D deficient may be a relevant contributor to an increased risk of falls [140,142].

Bone histology in osteomalacia shows an excessive accumulation of osteoid matrix with poor mineralization [135]. Collagen matrix is normally produced because osteoblast function is preserved, but the inadequate calcium–phosphate product in the extracellular space hinders matrix mineralization. Therefore, collagen matrix becomes gelatin-like and expands when exposed to water; this can occur below the periosteum leading to bone pain [143]. Another characteristic sign is proximal muscle weakness that results in difficulty in standing up and sometimes even in lifting the head due to severe weakness of shoulder girdle muscles. Patients usually complain of fatigue, which may be misdiagnosed with fibromyalgia, chronic fatigue syndrome or polymyalgia rheumatica [144].

### 4.3. Management of Vitamin D Deficiency

As mentioned, most of the vitamin D requirement is acquired by sun exposure. However, it is not reasonable to recommend a universal dose of sun exposure that can fit everyone, sufficient to obtain the indispensable annual requirement of vitamin D. In fact, a number of parameters come into play, including age, somatic characteristics, weather, time of exposure, seasonal period, etc. As shown in Table 5, diverse scientific societies and international agencies have identified daily requirements of vitamin D in conditions of minimum sun exposure and recommended doses for its integration based on the circulating levels of 25(OH)D.

Even if there is general agreement on the need for adequate levels of vitamin D for bones and general health among various scientific societies and international agencies, the correct method for restoring optimal levels of vitamin D is still debated. Therapy for vitamin D deficiency-related rickets and osteomalacia aims to relieve symptoms, repair bone strength, promote fracture healing, optimize bone response to antiresorptive and bone-forming treatments, and improve quality of life, all of which are generally accompanied with the correction of the biochemical abnormalities. As shown, there are no universal guidelines to undertake the therapy; hence, treatments are based on the clinician experience and the availability of diverse vitamin D preparations. A study examining 675 iliac crest biopsies from male and female patients did not find pathologic accumulation of osteoid in any patient with circulating 25(OH)D above 30 ng/mL [145]; thus, it is reasonable to suggest that the prescribed dose of vitamin D supplementation should ensure achieving this level of 25(OH)D in order to maintain skeletal health.

In obese patients with vitamin D deficiency, in patients with malabsorption, and in patients receiving medications interfering with vitamin D metabolism, the dosage of vitamin D therapy necessary to correct the deficit should be increased by 2- to 3-fold compared to normal weight persons, or without these pathologies [146]. In pregnant women, 4000 IU/day of cholecalciferol was effective in raising serum 25(OH)D concentrations in the range of 40 to 60 ng/mL, levels that have been associated with reduced risk for preeclampsia, premature births, and need for cesarean section [147]. Human breast milk, in essence, contains no vitamin D. However, vitamin D content of a mother’s milk is directly related to maternal vitamin D status, and if the woman was deficient during pregnancy, in the course of lactation—a period when the requirement is higher—her milk will be deficient, unless she takes higher doses of vitamin D. Because of this relative “deficiency,” maternal supplementation with 6400 IU vitamin D/day is recommended, which is effective in safely raising maternal circulating level of vitamin D, and that of her breast milk, warranting that it has sufficient vitamin D, in order to meet the infant’s requirement [148]. If not, the American Academy of Pediatrics and Endocrine Society recommended that the infant should receive 400 to 600 IU/day [7].

While cholecalciferol remains the most commonly disseminated form of vitamin D supplementation worldwide, other preparations are available for clinical use, which are not equipotent, as will be discussed below. Hence, it is vital to recognize the differences in order to select the most appropriate compound and dosage in an individual basis (Table 6).

The principal aim of the therapy is to replenish vitamin D stores, afterwards, patients continue on a maintenance dose. Since dietary sources are unlikely to be sufficient, especially for vegetarians and vegans, supplements are often necessary to properly correct vitamin D deficiency. Moreover, in conditions where sun exposure is inadequate, or cutaneous synthesis is decreased, e.g., in older adults, who are at a high risk of severe deficiency, prescribing vitamin D supplements is often necessary. The rational for supplying adequate amounts is that high serum PTH concentrations, even in patients with subclinical vitamin D deficiency, may contribute to bone fragility and falls in older adults. This secondary hyperparathyroidism can be effectively lessened by the administration of vitamin D supplements.

The most common forms of vitamin D supplements are cholecalciferol and ergocalciferol. A meta-analysis of seven RCTs directly comparing the effects of these two compounds on 25(OH)D levels showed that supplementation with cholecalciferol was more efficient than ergocalciferol, with a mean difference of 6 ng/mL in serum 25(OH)D increase [149]. Similar results were obtained in a later RCT comparing cholecalciferol with ergocalciferol in fortified foods [150]. The greatest difference was reported in trials using weekly or monthly vs. daily dosing. Nevertheless, there was significant heterogeneity among the studies included in the meta-analysis.

The dosage of vitamin D supplements necessary to effectively treat vitamin D deficiency is variable, since it depends on several factors, mainly linked to individual characteristics, such as the capacity of vitamin D absorption and of liver hydroxylation, as well as genetic unknown causes. The responsiveness also depends on the baseline levels of 25(OH)D. Several studies have calculated that, in a person with preserved absorption capacity, for each 100 IU of added cholecalciferol, serum 25(OH)D levels would increase approximately 0.7–1.0 ng/mL, with the greatest increase observed in patients with the lowest baseline 25(OH)D concentrations. The increase is not linear with cholecalciferol supplementation and declines when 25(OH)D levels reach values higher than 40 ng/mL [61,151,152,153]. There have been reports showing that the increase in serum 25(OH)D levels for a given dose of cholecalciferol tends to stabilize by the sixth week [154], and that it does not vary with age, at least up to 80 years of age [94,154,155,156]. Efficacy in treating vitamin D deficiency has been reported for various dosing regimens [157,158]. However, high loading doses are not recommended due to potential negative effects (see below toxicity section).

Another option to treat vitamin D deficiency is to use its metabolites, especially in conditions where there is abnormal liver or kidney function. The choice of preparation and dosage vary, according to the specific clinical condition. Calcifediol [25(OH)D] is useful in patients with liver disease as it does not require hepatic 25-hydroxylation. This compound can be also beneficial in patients with malabsorption because it is more hydrophilic than cholecalciferol or ergocalciferol, and the onset of action is faster. In patients with liver disease, vitamin D deficiency can be treated with 30 to 200 μ/day of calcifediol [159]. The faster absorption of calcifediol is explained because it occurs through the portal vein circulation in comparison with the more complex lymphatic pathway used by cholecalciferol. This transportation difference may (at least in part) explain the greater bioavailability [160]. The main determinant of the length of time a vitamin D metabolite remains in the circulation is its affinity to DBP [161]. The dissociation constant of this binding, which is different in calcifediol (Table 5), determines the free concentrations, which enables the molecule diffusion across the cell membrane and, thereby, the cellular activity. The different affinity contributes to the diverse circulating half-life of the metabolites, with cholecalciferol exhibiting a half-life of about two days, calcifediol of three weeks, and calcitriol of few hours [42]. Therefore, DBP sustains stable levels of vitamin D metabolites and regulates their bioavailability, activation, and reactivity of the target organs [162]. As opposed to cholecalciferol, calcifediol has been shown to have a linear absorption when administered in daily or weekly schedules. When calcifediol was administered in postmenopausal women for three months, 25(OH)D serum levels were raised without modifications in other parameters of mineral metabolism, and the magnitude of absolute percentage increase was similar for those with baseline levels below or above 20 ng/mL [163]. Because inhibition of liver cytochrome isoforms has been reported in uremia, calcifediol was suggested as useful in patients with chronic renal failure [164].

Supplementation with calcifediol has been reported in an efficient manner to correct poor vitamin D status in several studies [165,166,167,168,169,170,171,172,173,174,175,176,177,178] (Table 7). Even if the most common form of vitamin D supplementation used today is cholecalciferol, the usual recommended doses are frequently not able to rapidly correct vitamin D insufficiency, especially in severe cases. One pharmacokinetic study suggests that it takes approximately 68 days with 800 IU/day of cholecalciferol to achieve the optimal plateau level [173]. This time could be reduced by increasing the dose or using a high bolus-loading dose, with the purpose of reaching the recommended levels of 25(OH)D for skeletal and general health in a relatively short period of time [179]. Even if high doses of up to 10,000 IU/day are safe, in regards to hypercalciuria and hypercalcemia [180], the most recent guidelines recommend not to use them due to the possible adverse effects (see Toxicity section below). Some studies have also shown that high-bolus doses ≥100,000 IU of cholecalciferol significantly increased bone resorption markers in a dose-dependent manner [181,182].

Calcitriol, the active form of vitamin D [1,25(OH)_2_D], is useful in patients with calcitriol decreased synthesis and severe secondary hyperparathyroidism, due to chronic renal failure or in the genetic disease, type 1 vitamin D-dependent rickets [183]. It has a rapid onset of action and a half-life of only a few hours. Calcitriol is associated with a fairly high incidence of hypercalcemia; hence, serum calcium should be monitored carefully. During treatment with calcitriol in patients with renal failure, 25(OH)D serum levels are not indicative of the clinical vitamin D status [184].

Several RCTs with different designs (six double-blind RCTs and seven open-label RCTs) shown in Table 7 in chronological order, have been conducted, comparing the ability of calcifediol with that of cholecalciferol to increase serum 25(OH)D concentrations. Moreover, the studies using different dosages, single or multiple, were conducted in heterogeneous populations, and in general, included not a very high number of participants, and all reported that calcifediol was more potent than cholecalciferol (2–8 fold) and that its use resulted in a faster increase of 25(OH)D.

## 5. Vitamin D Excess/Toxicity

According to the National Academy of Medicine (former Institute of Medicine, IOM) Report in 2011, acute vitamin D toxicity is usually caused by doses of vitamin D above 10,000 IU/day, resulting in serum 25(OH)D concentrations over 150 ng/mL. Chronic vitamin D toxicity can potentially occur with administration of doses above 4000 IU/day for extended periods, likely resulting in 25(OH)D concentrations between 50 and 150 ng/mL [185]. Traditionally vitamin D toxicity is considered for 25(OH)D levels above which hypercalcemia is likely to occur. As shown in Table 4, all scientific societies and international agencies consider levels of circulating 25(OH)D above 100 ng/mL as “toxicity” or associated with excess adverse events, and some even consider that the risks start increasing for levels above 50 ng/mL [7,185,186]. Noteworthy, vitamin D toxicity is caused by supplements, not by diet or sun exposure. Vitamin D3 was shown to be superbly sensitive to sunlight and, once formed in the skin, exposure to sunlight resulted in its rapid photodegradation to a variety of photoproducts, including 5,6-transvitamin D3, suprasterol I, and suprasterol II [27]. This helps explain why vitamin D toxicity from exposure to solar UV radiation does not occur, and may be relevant to public health messages on safe sun exposure; hence, short and recurrent periods of sun exposure are preferable to long exposure in order to attain vitamin D production, while minimizing UV-induced DNA damage [187].

Vitamin D toxicity should be taken in consideration because it can be potentially serious. Although vitamin D toxicity has been generally considered infrequent, in recent years, with the escalation of uncontrolled over-the-counter vitamin D use and eventual inappropriate prescriptions, most likely in combination with the consumption of various fortified foods, the cases of vitamin D toxicity have increased remarkably. However, the numbers are variable in different world regions. In a study from Minnesota, the age- and sex-adjusted incidence of 25(OH)D above 50 ng/mL increased from 9 to 233 cases per 100,000 person–years from 2002 to 2011, with the greatest increase in women and those older than 65 years old, without an apparent corresponding increase in acute clinical toxicity [188]. Another study from the U.S. showed that only 27 of 60,237 25(OH)D tests had values of 25(OH)D concentrations above 150 ng/mL [189]. Conversely, a study from Pakistan involving 2249 children showed that, although 64% of children had serum 25(OH)D concentrations below 30 ng/mL, 9.8% and 3.2% of children had concentrations above 80 and 150 ng/mL, respectively, much higher that the U.S. studies [190]. This highlights the problematic coexistence of vitamin D insufficiency and toxicity.

A retrospective analysis of data from the U.S. National Poison Data System reported an increase in toxic exposure to vitamin D from a mean of 196 cases per year from 2000 to 2005, to a mean of 4535 exposures per year from 2005 to 2011, considering that the total exposures were remarkably greater in that period. There were no fatalities, while serious medical outcomes (major or moderate) ranged from 2 patients/year to 22 patients/year [191]. Therefore, in spite of the enormous increase in number of exposures, the severe outcomes were rare. Nevertheless, it is essential to avoid excess administration of vitamin D. Even if single fortified foods do not contain large amounts of vitamin D, when combined with other fortified foods and/or supplements, the risk of toxicity increases.

Hypercalcemia can produce gastrointestinal symptoms (e.g., anorexia, nausea, vomiting, constipation), as well as weakness and fatigue. In severe cases, it can produce polyuria, polydipsia, renal failure, ectopic calcifications, as well as depression, confusion, stupor, or coma. Persistent hypercalcemia may lead to bone pain and urolithiasis. Hence, hypercalciuria, which occurs at much lower levels of 25(OH)D, may be a sign of vitamin D toxicity, especially when prescribed together with calcium supplements, as frequently occurs in postmenopausal women. In a RCT, 163 Caucasian women aged 57 to 90 years with baseline deficiency [25(OH)D <20 ng/mL] received oral vitamin D at doses ranging from 400 to 4800 IU/day, and calcium citrate was added to the diet, to achieve 1200 mg/day. After three months, hypercalcemia (>10.2 mg/dL) occurred in 8.8% of participants, while hypercalciuria (>300 mg/day) occurred in 30.6% of participants. Hypercalciuria was transient in half of the group and recurrent in the other half, and it was also common in the placebo group; hence, it was not clear whether hypercalciuria and hypercalcemia were caused by calcium, vitamin D, or both [153]. Most studies agree with the notion that doses up to 4000 IU/day (or its equivalent monthly) are probably safe in the majority of people [192,193]. A 3-year, double-blind, RCT from Canada assessed the dose-dependent effect of vitamin D supplementation on volumetric BMD and strength in 311 community-dwelling healthy adults, aged 55 to 70 years, with baseline 25(OH)D levels of 12–50 ng/mL (mean of 31 ng/mL). Participants were randomized to receive vitamin D3 at 400, 4000, or 10,000 IU/day for three years. Calcium supplementation was added to the diet to achieve 1200 mg/day. As expected, 25(OH)D levels increased in a dose-dependent manner. However, treatment with vitamin D3 for 3 years at doses of 4000 IU/day or 10,000 IU/day vs. 400 IU/day resulted in a statistically significant reduction in radial volumetric BMD, without significant modifications in bone strength at the radius or tibia. The authors concluded that the results did not support the use of high-dose vitamin D supplementation (≥4000 IU/day) for bone health, and that potential, harmful effects were uncertain, and warranted further study [194].

An important outcome, fall risk, has been the focus of discussion, according to some available studies [5,169,195]. A seminal double-blind, RCT of 2256 community-dwelling women, aged 70 years or older, at high risk of fracture, was published in 2010 by Sanders et al. Participants were randomly assigned to receive 500,000 IU of cholecalciferol or placebo during autumn–winter for 3–5 years. There was an increased risk of falls and fractures in the women who received cholecalciferol treatments, associated with levels of 25(OH)D higher than 45 ng/mL, with the risk particularly higher in the first three months after treatment [5]. In a 12-month double blind RCT, older women (mean age 66 years) with baseline 25(OH)D <20 ng/mL received seven different daily oral doses of vitamin D or placebo. The results showed that vitamin D followed a U-shaped curve effect on falls, with no decrease in falls at low vitamin D daily doses (400, 800 IU), a significant decrease at medium daily doses (1600, 2400, 3200 IU), and no decrease at high daily doses (4000, 4800 IU) vs. placebo. Fall rates at the high doses were significantly increased compared to medium doses (OR 5.6; 95% CI: 2.1–14.8) [195]. In another double-blind RCT conducted in Switzerland, 200 community-dwelling men and women, aged ≥70 years with a prior fall received, for one year, a monthly supplementation with 24,000 IU of vitamin D3, or 60,000 IU of vitamin D3, or 24,000 IU of vitamin D3 plus 300 μg of calcifediol. Even if higher monthly doses of vitamin D were effective in reaching a threshold of at least 30 ng/mL of 25(OH)D, they had no benefit on lower extremity functions, and were associated with an increased risk of falls, compared with 24,000 IU. It is noteworthy that 42% of participants were vitamin D replete at baseline (>20 ng/mL) and only 13% were severely deficient (<10 ng/mL) [169].

## 6. Conclusions

Even if vitamin D/hormone D is essential for bone health, its deficiency is still prevalent worldwide across all ages, sexes, ethnicities, and socioeconomic conditions, making it a major public health problem. Even nutritional rickets, a fully curable disease, affects a significant number of infants and children globally. Thus, a global effort is needed to eradicate this devastating and treatable condition. There are several gaps in knowledge about vitamin D deficiency and its treatment, which should be addressed to manage this important public health concern: (i) there is a lack of proper standardization in the analytical methods used to quantify 25(OH)D concentrations, which remain the universally used marker of deficiency. This, together with other variables, contributes to the non-uniform indications and thresholds of guidelines from scientific societies and international agencies, based on heterogeneous study results. (ii) There is lack of data on acceptable 25(OH)D concentrations in infants, children, pregnant and lactating women, as well as in certain ethnic groups. Likewise, little attention is devoted to the prevalent vitamin D deficiency among bariatric surgery patients and athletes. (iii) Many people at high risk of vitamin D deficiency (e.g., older adults, obese, persons with diabetes, CKD, or malabsorption) are not adequately evaluated and treated. Conversely, growing interest among lay individuals has contributed to a wide, empirical use of vitamin D supplements, without monitoring, and for prolonged periods of time, in people at low risk who probably do not need them (this may increase the risk of toxicity). (iv) Vitamin D deficiency can be corrected with different compounds that have different pharmacokinetic characteristics and potency to increase 25(OH)D serum concentrations. Thus, it is crucial to recognize the differences to choose the most appropriate compound and dosage at an individual basis. v) A number of observational prospective studies have shown significant associations of low 25(OH)D concentrations with an increased risk of multiple health outcomes, but RCTs and meta-analyses testing the efficacy of vitamin D supplementation have frequently failed to support any benefit, besides a reduction in mortality. Among several possible explanations for these results, it is crucial to recognize that most participants in the trials did not have vitamin D deficiency.

These uncertainties should not downplay the need to face the fundamental problem of vitamin D deficiency, and recognize the crucial role of maintaining adequate vitamin D status to improve bone health and overall health.

## Figures and Tables

**Table 1:** Content of vitamin D in some foods.

Food	mcg per Serving	IU per Serving
Cod liver oil, 1 tablespoon	34.0	1360
Trout (rainbow), cooked, 3 ounces	16.2	645
Salmon (sockeye), cooked, 3 ounces	14.2	570
Mushrooms, raw, exposed to UV light, ½ cup	9.2	366
Sardines, canned in oil, drained, 2 sardines	1.2	46
Egg, 1 large, scrambled *	1.1	44
Liver, beef, braised, 3 ounces	1.0	42
Tuna fish, canned in water, drained, 3 ounces	1.0	40
Cheese, cheddar, 1 ounce	0.3	12
Mushrooms, portabella, raw, diced, ½ cup	0.1	4
Chicken breast, roasted, 3 ounces	0.1	4
Beef, ground, 90% lean, broiled, 3 ounces	traces	1.7
Broccoli, raw, chopped, ½ cup	0	0
Carrots, raw, chopped, ½ cup	0	0
Almonds, dry roasted, 1 ounce	0	0
Apple, large	0	0
Banana, large	0	0
Rice, brown, long-grain, cooked, 1 cup	0	0
Whole wheat bread, 1 slice	0	0
Lentils, boiled, ½ cup	0	0
Sunflower seeds, roasted, ½ cup	0	0
Edamame, shelled, cooked, ½ cup	0	0
*Fortified Foods*		
Milk, 2% milkfat, vitamin D fortified, 1 cup	2.9	120
Soy, almond, and oat milks, vitamin D fortified, various brands, 1 cup	2.5–3.6	100–144

mcg: micrograms; IU: international units. Data from Vitamin D Fact Sheet for Health Professionals, National Institute of Health (https://ods.od.nih.gov/factsheets/VitaminD-HealthProfessional/#h3 accessed on 25 March 2021). * Vitamin D is in the yolk.

**Table 2:** Main biological actions of vitamin D/hormone D.

Calcium and phosphorus homeostasis: ○ Increased intestinal calcium absorption and synthesis of the intestinal calcium transporter; ○ Increased intestinal absorption of phosphorus; ○ Increased renal reabsorption of calcium and phosphorus; ○ Induction of mature osteoblasts differentiation from precursors; ○ Stimulation of bone resorption. Immunomodulatory effects: ○ Induction of monocytes differentiation to macrophages; ○ Increased rate of phagocytosis; ○ Increased production of lysosomal enzymes; ○ Decreased interleukin-2 production; ○ Increased interleukin-10 production. Antitumor effect: ○ Induction of cell differentiation; ○ Increased apoptosis of neoplastic cells. Cardiovascular effect: ○ Reduction of plasma renin activity and angiotensin II levels.

**Table 3:** Drug interactions with vitamin D/hormone D.

Interference with vitamin D absorption: ○ Bile acid sequestrants (cholestyramine); ○ Lipase inhibitors (orlistat). Interference with vitamin D metabolism: ○ Antiepileptic drugs (phenobarbital, phenytoin); ○ Corticosteroids; ○ Statins; ○ Antimicrobials (rifampicin, isoniazid, hydroxychloroquine, immunosuppressive agents (cyclosporine, tacrolimus); ○ Chemotherapeutic agents; ○ Highly active antiretroviral agents; ○ Histamine H2-receptor antagonists; Interaction that may induce side effects: ○ Thiazides (risk of hypercalcemia due to calcium-sparing effect).

**Table 4:** Diverse thresholds of serum vitamin D [25(OH)D] for the definition of sufficiency, insufficiency, or deficiency proposed by diverse scientific societies and international agencies.

25(OH)D ng/mL	NAM/NIH	ES	NOS	SACN	AGS *	ESE
<10	deficiency	deficiency	deficiency	deficiency	deficiency	deficiency
10–20	inadequacy risk	deficiency	inadequacy risk	sufficient	deficiency	deficiency
20–30	sufficiency	insufficiency	sufficiency	sufficient	deficiency risk	insufficiency
30–50	sufficiency	desirable concentration	sufficiency	sufficient	minimal acceptable concentration	sufficiency
50–100	possible excess adverse events	desirable concentration			possible onset of toxicity	
100–150	possible excess adverse events				possible onset of toxicity	
>150					toxicity	

NAM: National Academy of Medicine (former Institute of Medicine, IOM), USA; NIH: National Institute of Health, USA; ES: Endocrine Society, USA; NOS: National Osteoporosis Society, UK; SACN: Scientific Advisory Committee on Nutrition, UK; American Geriatrics Society, USA; ESE: European Society of Endocrinology. * Values applicable to old age.

**Table 5:** Diverse therapeutic dosage of vitamin D recommended by scientific societies and international agencies.

25(OH)D ng/mL	NAM/NIH	ES	NOS	SACN-PHE	AGS ^4^
		Initial Dose ^1^—Maintenance ^2^	Initial Dose ^3^—Maintenance ^2^		
<10	600 IU ^5^	400,000 IU 1500–2000 IU ^6,7^	300,000 IU 800–2000 IU	–	4000 IU ^11^
10–20	600 IU ^5^	400,000 IU 1500–2000 IU ^6,7^	400 IU	400 IU ^10^	4000 IU ^11^
20–30	600 IU ^5^	1500–2000 IU ^6,8,9^	400 IU	400 IU ^10^	4000 IU ^11^
30–50	600 IU ^5^	1500–2000 IU ^8,9^	400 IU	400 IU ^10^	4000 IU ^11^
50–100	–	1500–2000 IU ^8,9^	–	–	–
>100	–	–	–	–	–

NAM: National Academy of Medicine (former Institute of Medicine, IOM), USA; NIH: National Institute of Health, USA; ES: Endocrine Society, USA; NOS: National Osteoporosis Society, UK; SACN: Scientific Advisory Committee on Nutrition, UK; American Geriatrics Society, USA. ^1^ The initial or attack does should be given within 8 weeks. ^2^ Daily. ^3^ To be given weekly or daily; initial or attack dose is necessary only in cases where correction of vitamin d levels is considered urgent. ^4^ Reference for older adults or adults at risk. ^5^ 800 IU in older adults (aged >70 years). ^6^ For obese patients, 336,000 to 560,000 IU as initial dose and 3000 to 6000 IU for maintenance. ^7^ For pediatric patients, 50,000 IU weekly as initial dose; for maintenance, 400–1000 IU daily for children less than one year old or 600–1000 IU daily for children and adolescents aged 1–18 years. ^8^ 400–1000 IU daily for children aged 6–12 months or 600–1000 IU daily for children and adolescents aged 1–18 years. ^9^ During lactation, the dose to be assumed by the mother in case the child does not receive 400 IU/day is 4000–6000 IU. ^10^ Supplementation is recommended in groups at risk, such as pregnant or lactating women, age >65 years, dark skin, independently of serum levels of vitamin D (for these groups the routine control of vitamin D levels is not recommended). ^11^ Daily dose will be assessed case-by-case on the basis of nutritional intake, season, eventual presence of obesity, and skin pigmentation.

**Table 6:** Chemical structure and pharmacokinetic characteristics of vitamin D compounds and activated forms.

	Ergocalciferol	Cholecalciferol	Calcifediol (or Calcidiol)	Calcitriol
Chemical Structure	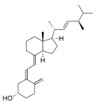	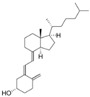	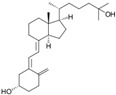	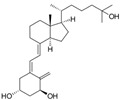
Absorption	Intestine (bile required)	Intestine (bile required)	Intestine, readily absorbed *	Intestine, readily absorbed *
DBP dissociation constant	10^−7^	10^−7^	10^−9^	10^−7^
Volume of distribution	very limited in plasma compartment; rapidly stored in fat tissue	very limited in plasma compartment; rapidly stored in fat tissue	larger than plasma volume	plasma compartment
Tissue distribution for long-term	adipose tissue, muscle	adipose tissue, muscle	blood, adipose tissue, muscle	blood and tissues
Circulating half-life	2 days	2 days	3 weeks	4–8 h
Functional half-life	2–3 months	≤2 months	2–3 months	4–8 h

* Faster absorption than ergocalciferol and cholecalciferol. DBP: vitamin D binding protein.

**Table 7:** Summary of results from studies comparing supplementation with cholecalciferol vs. calcifediol.

Authors/Country	Year	Number and Type of Participants	Study Design	Cholecalciferol/Calcifediol Dose	Duration	Summary of Results
Corrado et al. Italy [165]	2021	107 postmenopausal women (mean age 60.8 ± 6.5 y)	Open-label RCT	–cholecalciferol: 100,000 IU single dose or 100,000 IU/month or 7000 IU/week –calcifediol: 7000 IU/week	6 months	Weekly calcifediol and cholecalciferol induced a greater and faster increase of serum 25(OH)D vs. monthly or single-dose cholecalciferol administration; 25(OH)D increase was associated with improved lower limbs muscle function. Supplementation with calcifediol was more effective and faster vs. cholecalciferol in increasing 25(OH)D serum levels and was associated with a greater improvement of muscular function.
Ruggiero et al. Italy [166]	2019	67 community-dwelling women and men, aged >75 y	Open-label RCT	–calcifediol: 150 μg/week –cholecalciferol: 150 μg/week	7 months	Supplementation with calcifediol and cholecalciferol were associated with significant increasing serum levels of 25(OH)D and 1,25(OH)_2_D in oldest–old persons, with a steeper rise and faster recovery of acceptable iPTH levels for those on calcifediol; after adjustment for iPTH levels the differences disappeared. Both supplementations were associated with a decreasing trend of iPTH and CRP. Polypharmacy and low muscle strength weaken the recovery of adequate 25(OH)D serum levels.
Vaes et al. Netherlands [167]	2018	59 men and women aged >65 y	Double-blind RCT	–calcifediol: 5, 10 or 15 μg/day –cholecalciferol: 20 μg/day	24 weeks	Supplementation with 20 μg/day of cholecalciferol increased 25(OH)D3 concentrations towards 28 ng/mL within 16 weeks. Supplementation with 10 or 15 μg/day of calcifediol increased 25(OH)D3 levels >28 ng/mL/L in 8 and 4 weeks, respectively. Steady state was achieved from week 12 onwards with serum 25(OH)D3 levels stabilizing between 84 and 89 nmol/L (33.6 and 35.6 ng/mL) in the 10 μg/day calcifediol group. No cases of hypercalcemia occurred in any treatment during the study period.
Shieh et al. USA [168]	2017	35 aged ≥18 y with 25(OH)D <20 ng/mL, from a multiethnic cohort	Open-label RCT	–calcifediol: 20 μg/day –cholecalciferol: 60 μg/day	16 weeks	Significant higher and faster increment of total and free 25(OH)D with calcifediol vs. cholecaciferol (total: +25.5 vs. +13.8 ng/mL; free: +6.6 vs. +3.5 pg/mL). By 4 weeks, 87.5% of calcifediol treated participants had total 25(OH)D levels ≥30 ng/mL, vs. 23.1% of cholecalciferol treated participants. Conclusions: calcifediol increased total and free 25(OH)D levels more rapidly than cholecalciferol, regardless of race/ethnicity. Free and total 25(OH)D were similarly associated with change in PTH.
Bischoff-Ferrari et al. Switzerland [169]	2016	200 community-dwelling men and women (67%) aged ≥70 y with a prior fall; 58% were vitamin D deficient (<20 ng/mL) at baseline	Double-blind RCT	–Group 1: cholecalciferol 24,000 IU/month –Group 2: cholecalciferol 60,000 IU/month –Group 3: cholecalciferol 24,000 IU/month plus calcifediol 300 μg/month	12 months	Participants in Group 3 vs. Group 1 were significantly more likely to achieve 25(OH)D levels of at least 30 ng/mL. Lower extremity function did not differ among the treatment groups. The incidence of falls was higher for Groups 2 and 3 vs. Group 1.
Navarro-Valverde et al. Spain [170]	2016	40 post-menopausal women (in 4 groups), mean age 67 ± y, deficient in vitamin D [mean 25(OH)D <15 ng/mL]	Open-label RCT	–Group 1: cholecalciferol 20 μg/day –Group 2: calcifediol 20 μg/day –Group 3: calcifediol 0.266 μg/week –Group 4: calcifediol 0.266 μg/two weeks	12 months	Calcifediol was significantly faster and 3–6 times more potent to obtain serum levels of 25(OH)D in the medium to long term. The authors concluded that both metabolites are not equipotent and that the therapeutic prescription guidelines should consider the differences to avoid over-dosage of calcifediol.
Meyer et al. Switzerland [171]	2015	20 post-menopausal women, mean age 61.5 ± 7.2 y, 25(OH)D between 8 and 24 ng/mL [mean 25(OH)D 13.2 ng/mL]	Double-blind RCT	–calcifediol: 20 μg/day –cholecalciferol: 20 μg/day	4 months	Increase in 25(OH)D levels was significantly higher in the calcifediol group vs. cholecalciferol group (to a mean of 69.3 ± 9.5 ng/mL vs. 30.5 ± 5.0 ng/mL, respectively). Calcifediol vs. cholecalciferol improved gait speed by 18% among these young postmenopausal women, after adjustments for baseline gait speed, age, and BMI. Changes in 25(OH)D blood levels over time were significantly correlated with improvement in gait speed. No effect could be demonstrated for trunk sway.
Catalano et al. Italy [172]	2015	57 postmenopausal women at low risk of fracture, on atorvastatin treatment, mean age 59 ± 6.7 y, 25(OH)D <30 ng/mL [mean 25(OH)D 13.2 ng/mL]	Open-label RCT	–calcifediol: 140 μg/week –cholecalciferol: 140 μg/week	24 weeks	25(OH)D increased significantly in both groups with higher levels in participants receiving calcifediol vs. cholecalciferol. Only in the calcifediol group, a significant reduction of LDL-C and an increase of HDL-C were observed, after adjustment for age, and baseline BMI, 25(OH)D and lipid levels. The percent changes in 25(OH)D levels were significantly associated with the variations of LDL-C but not with HDL-C levels.
Jetter et al. Switzerland [173]	2014	35 healthy females aged 50–70 y, 25(OH)D between 8 and 24 ng/mL	Double-blind RCT	–calcifediol: 20 μg/day or 140 μg/week or both for 15 weeks or a single bolus of 140 μg –cholecalciferol: 20 μg/day or 140 μg/week or both for 15 weeks or a single bolus of 140 μg –calcifediol single bolus of 140 μg plus cholecalciferol single bolus of 140 μg	15 weeks or single bolus	All women in the daily and weekly calcifediol groups achieved 25(OH)D3 concentrations >30 ng/mL (mean, 16.8 days), but only 70% in the cholecalciferol daily or weekly groups reached this concentration (mean, 68.4 days). A single dose of 140 μg calcifediol led to 117% higher 25(OH)D3 AUC0-96h values than 140 μg vitamin D3, while the simultaneous intake of both did not further increase exposure. The authors concluded that calcifediol given daily, weekly, or as a single bolus is about 2–3 times more potent in increasing plasma 25(OH)D3 concentrations vs. cholecalciferol, and concentrations of 30 ng/mL were reached more rapidly with calcifediol.
Bischoff-Ferrari et al. Switzerland [174]	2012	20 healthy postmenopausal women, with a mean 25(OH)D level of 13.2 ± 3.9 ng/mL and a mean age of 61.5 ± 7.2 y	Double-blind RCT	–calcifediol: 20 μg/day –cholecalciferol: 20 μg/day	4 months	Mean 25(OH)D levels increased rapidly to 69.5 ng/mL in the calcifediol group and to 31.0 ng/mL with a slow increase in the cholecalciferol group. All analyses were adjusted for baseline measurement, age, and BMI. Therapy with calcifediol vs. cholecalciferol had a significant 2.8-fold increased odds of maintained or improved lower extremity function, and a 5.7-mmHg significant decrease in SBP. Both types of vitamin D contributed to a decrease in five out of seven markers of innate immunity, significantly more pronounced with calcifediol for eotaxin, IL-12, MCP-1, and MIP-1 beta. There were no cases of hypercalcemia at any time point.
Cashman et al. Ireland [175]	2012	56 healthy, free-living adults aged ≥50 y	Double-blind RCT	–calcifediol: 7 or 20 μg/day –cholecalciferol: 20 μg/day	10 weeks	The mean increases (per μg of vitamin D compound) in serum 25(OH)D concentrations were 0.96 ± 0.62, 4.02 ± 1.27, and 4.77 ± 1.04 nmol/L for 20 μg/day of cholecalciferol and 7- and 20 μg/day of calcifediol, respectively. A comparison of the 7- and 20-μg of calcifediol groups with the 20 μg of cholecalciferol group yielded conversion factors of 4.2 and 5, respectively. There was no effect on serum calcium concentrations and no incidence of hypercalcemia. The authors concluded that each μg of calcifediol was about 5 times more effective in raising serum 25(OH)D in older adults in winter than an equivalent amount of cholecalciferol.
Rossini et al. Italy [178]	2005	271 postmenopausal women with osteopenia or osteoporosis with hypovitaminosis D	Open-label RCT	–calcifediol: 100 μg/week –cholecalciferol: 20–22 μg/day	12 months	The compliance to the weekly calcifediol was over 90% and led to serum levels of 25(OH)D, similar to those obtained with daily cholecalciferol. The potency of calcifediol vs. cholecalciferol in increasing 25(OH)D was 1.66 fold, but the study aimed to evaluate compliance, not efficacy.
Barger-Lux et al. USA [176]	1998	116 healthy men with usual milk consumption of ≤0.47 L/day, mean age of 28 ± 4 y, mean serum 25(OH)D of 26.8 ± 10 ng/mL from January to April	Open-label RCT	–Cholecalciferol 25, 250 or 1250 μg/day –Calcifediol 10, 20 or 50 μg/day –Calcitriol 0.5, 1.0 or 2.0 μg/day	8 weeks (group 1) 4 weeks (group 2) 2 weeks (group 3)	In participants treated with cholecalciferol serum 25(OH)D increased by 11.6, 58.4, and 257.2 ng/mL for the three dosage groups, respectively. Treatment with calcifediol increased circulating 25(OH)D by 16, 30.4, and 82.4 ng/mL, respectively. Treatment with calcitriol increased circulating 1,25(OH)2D by 10, 46, and 60 pmol/L, respectively. Slopes calculated from these data allowed the following estimates of mean treatment effects for typical dosage units in healthy 70-kg adults: an 8-week course of cholecalciferol at 10 μg/day would raise serum 25(OH)D by 4.4 ng/mL and a 4-week course of calcifediol at 20 μg/day would raise serum 25(OH)D by 37.6 ng/mL (potency of calcifediol vs. cholecalciferol in increasing 25(OH)D was 3.3–3.5 fold at a low dose and 7–8 fold for the highest dose of both compounds).
Stamp et al. UK [177]	1977	200 participants	Clinical practice		5 years	Ten times more cholecalciferol/ergocalciferol than calcifediol was required to produce equivalent plasma 25(OH)D concentration. The authors conclude that these data indirectly measure the superior therapeutic potency of calcifediol and the possible usefulness in patients with reduced 25-hydroxylation of vitamin D, or reduced solar exposure. Limitations of this study include: inclusion of patients with metabolic bone diseases; lack of homogeneity among the groups; use of ergocalciferol and cholecalciferol interchangeably without separating the results obtained by each of them; differences in duration of treatments of the diverse compounds.

AUC0-96h: area under the concentration-time curve; BMI: body mass index; CRP: C reactive protein; IL: interleukin; iPTH: intact parathyroid hormone; HDL-C: high-density lipoprotein cholesterol; LDL-C: low-density lipoprotein cholesterol; MCP-1: monocyte chemotactic protein-1; MIP-1 beta: macrophage inflammatory protein-1 beta; RCT: randomized controlled trial; SBP: systolic blood pressure; y: years.
